# Polyandry Has No Detectable Mortality Cost in Female Mammals

**DOI:** 10.1371/journal.pone.0066670

**Published:** 2013-06-18

**Authors:** Jean-François Lemaître, Jean-Michel Gaillard

**Affiliations:** Université de Lyon, Lyon; Université Lyon 1; CNRS UMR5558, Laboratoire de Biométrie et Biologie Evolutive, Villeurbanne, France; German Primate Centre, Germany

## Abstract

In several taxonomic groups, females mate with several males during a single reproductive cycle. Although there is evidence that polyandry provides some benefits to females, it often involves mortality costs. However, empirical evidences of mortality costs of polyandry have so far been reported only in invertebrates. Whether polyandry has mortality costs in vertebrates is currently unknown. In the present study, we aimed to fill the gap by investigating the relationships between the level of polyandry (measured either by male relative testes mass or the percentage of multiple paternities) and female patterns of mortality across mammals. While we found that the two metrics of female mortality co-varied with pace of life, we did not find any evidence that polyandry leads to either decreased median lifespan or increased aging rate in mammals. We discuss such an absence of detectable mortality costs of polyandry in female mammals in light of recent advances in the study of mammalian reproductive biology and life-history tactics.

## Introduction

In the last decade, several reviews have compiled convincing evidence that polyandry (i.e., when females mate with more than one male during a single reproductive cycle [1]) provides a large range of benefits to females [2]–[6]. For example, females can gain either direct benefits when nutritional gifts enhance female fertility or genetic benefits when multiple mating increases offspring quality [4], [6]. However, polyandry is also associated with various costs that might limit its occurrence [7], [8]. To date, most studies on costs of polyandry have focused on longevity costs in invertebrates [9], [10] for which increased mating rates (with the same or different males) often leads to elevated mortality risks through various pathways. For instance, polyandry increases the number of sexual contacts for females and ultimately the risk of contracting sexually transmitted diseases [11]. Polyandrous females can also suffer from higher mortality than monandrous females when high mating rates increase sensitivity to environmental risks (e.g. predation risk, environmental stochasticity) [12], [13]. For example in almond moths (*Cadra cautella*), the longevity cost of water deprivation is stronger for females that mate twice compared to females that mate only once [14]. Additionally, sperm competition (when sperm from two males or more compete to fertilize a set of ova [15]) faced by males in species with polyandrous females has driven the evolution of physiological, behavioural and anatomical traits in males that can jeopardize female survival [16], [17]. One striking example is the complex male genitalia found in insects or mammals [18], [19]. In these species, males often have penises that enhance their reproductive success in a sperm competition context but that also deeply damage female reproductive tracts [20]. Finally, males from polyandrous species have developed larger seminal vesicles [21], [22] whose by-products can trigger female immunosuppression, making them more susceptible to pathogens [23]. For example, a high exposure to seminal fluid products subsequent to an elevated number of mating increases death rate in *Drosophila melanogaster* [24].

Unfortunately, studies of longevity costs of polyandry are currently restricted to invertebrates [9], [10], [14], [25], [26] probably because it is easier to monitor matings and survival parameters in these species. Therefore, although polyandry occurs in all groups of vertebrates [1], it remains unknown whether longevity costs of polyandry occur in these taxa [6]. Moreover, studies in invertebrates have been often focused on maximum longevity although this measure does not fully account for the age-specific increase in probability of death [27]. Contrary to measures of aging, maximum longevity is also strongly dependent on sample size [28], [29] and refers to just one event in one individual and therefore may not be representative of the species as a whole [30], [31]. In mammals, individuals can suffer from various types of elevated physiological deterioration with age (e.g. aging in immune function in female Soay sheep, [32]), and the risk of dying during or following a mating through sexually transmitted disease, lethal injuries or predation is therefore likely to increase with age in females. The aging rate should thus provide a relevant metric to assess the relationship between mortality costs and polyandry.

In this study, we conduct a novel inter-specific analysis to investigate the relationships between polyandry and female mortality patterns in mammals using both the median lifespan (a measure of adult mortality less sensitive to sample size than maximum longevity) and the aging rate (a measure of the increase in mortality rate with age). In mammals, polyandry is widespread [33] and within species females show variability in their number of mates [34]. Similarly to other taxa, polyandry has evolved in mammals through various benefits conferred to females such as a higher reproductive success for offspring [35] or a reduced risk of infanticide by males [36]. However, quantifying the species-specific level of polyandry in mammals is far from an easy task. For instance, the mating system of primates is often based on observations performed on male social behaviour rather than on the direct number of female sexual partners [37]. Likewise, most species of large herbivores are simply classified as being polygynous, whereas they show a variable level of female polyandry [38]. To account for these problems we used the relative testes mass to measure the level of female polyandry. Indeed, there is now widespread evidence that the female propensity to mate repeatedly has strongly selected for an increase in testes mass (relative to body mass) within and across all mammalian groups [22], [37], [39], [40]. In addition, we used the proportion of multiple sired litters as a surrogate of polyandry when available [41]. We predicted that mortality costs associated with polyandry should increase with the level of polyandry. We thus expected the median lifespan to decrease and the aging rate to increase with either the relative testes mass and the proportion of multiple sired litters.

## Materials and Methods

### Dataset

We started our protocol search by looking for the maximum number of published studies with mammalian female demographic data from wild populations to compute female aging rate. We first gathered information on age-specific female mortality using the keywords ‘life table’ in the topic windows of *Web of Knowledge*. Then, we focused our protocol search on papers citing the two following ‘classics’: Deevey (1947, [42]) and Caughley (1966, [43]). These two highly cited papers (773 and 342 citations, respectively, on 15^th^ February 2013) were the first reviews of mammalian age-specific survival estimates and are likely to have been quoted by any mammalian study containing age-specific demographic data. In some studies, age-specific information was presented graphically only. Therefore, we extracted when possible the required data using OOodigitizer version 0.3.1., an Open Office extension (http://extensions.services.openoffice.org/) enabling extraction of coordinates from graphs. Finally, once age-specific survival data were gathered, we modelled survival for each species as a function of age using generalized additive models (GAM) [44] following the GAM procedure from the gam package [45]. This method allows the age at which survival starts to decrease to be estimated, and thereby the onset of senescence to be identified. Then we measured the aging rate of a given species as the slope of the linear regression of survival on age from the age at the onset of senescence onwards [46]. We also computed the female median lifespan as the age at which 50% of an initial cohort are still alive. As juvenile survival is highly variable between birth and 1 year of age [47], we used cohorts of individuals from 1 year of age onwards (i.e., cohort initialized at 1 year of age).

To assess the level of female polyandry, we extracted data on paired testes mass (measured without epididymis) and male body mass from individuals in breeding condition using published comparative reviews [48]. The allometric relationship between testes mass and body mass was linear (*N* = 51; β = 0.68±0.06; *t* = 10.98; *P*<0.0001), with no evidence for non-linearity. Data on percentage of multiple paternity and litter size were obtained from Soulsbury (2010, [41]). In absence of robust behavioural observations, the use of these two metrics (relative testes mass and percentage of multiple paternity) currently constitutes the most accurate method to assess the level of polyandry for each mammalian species [39]–[46].

In mammals both lifespan and aging rate of a given species are correlated with its pace of life [49], [50]. Indeed, species can be ranked along a slow-fast continuum where slow species are long-lived with a low aging rate and fast species are short-lived with a steep aging rate [46]. To avoid possible confonding effects of the pace of life, we thus corrected for the species ranking along the slow-fast continuum. We used age at first reproduction to correct for between-species differences in the pace of life because it provides the best surrogate in absence of generation time estimates [51]. We collected data on age at first reproduction from Wooton [52] and supplemented this information using more specific sources when required. Finally to avoid any multi-collinearity issues in our analyses, we checked that the pace of life was not correlated with the level of polyandry in mammals. This was revealed by the absence of a statistically significant relationship between relative testes mass and age at first reproduction (Table 1) and by the absence of a statistically significant relationship between the percentage of paternity and the age at first reproduction (pgls model with age at first reproduction (log-transformed) as a dependent variable (pgls: n = 17, adjusted R^2^ = 0.10, slope of −0.33±0.19, *t* = −1.70, *P* = 0.11).

**Table 1:** Parameter estimates from the model explaining variation in age at first reproduction by relative testes mass (corrected for body mass).

	Estimate ± SE	*t*	P
Testes mass	0.05±0.06	0.75	0.46
Body mass	0.20±0.05	3.92	<0.001

Relative testes mass is not statistically correlated with the age at first reproduction from a sample of 51 mammalian species. The adjusted R^2^ is equal to 0.54 and the lambda value is equal to 0.68. All variables are log-transformed.

Overall, we gathered information on female aging rate and testes mass for 51 mammalian species (19 Artiodactyla, 15 Carnivora, 7 Primates, 3 Rodentia, 3 Perissodactyla, 2 Proboscidae, 1 Erinaceomorpha and 1 Soricomorpha). Unfortunately, for three species (American mink, *Mustela vison*; Mandrill, *Mandrillus sphinx* and Indian rhinoceros, *Rhinoceros unicornis*) available life tables prevented us from calculating a reliable measure of median lifespan and we removed these species from the median lifespan analysis (see below). Data on proportion of multiple paternities was limited and available only for 17 species within our full dataset (10 Carnivora, 5 Artiodactyla and 2 Rodentia). All data and associated references are provided in the supplementary information (Table S1, S2 and S3).

### Statistical Analysis

We controlled for non-independence among species due to shared ancestry [53]. We first constructed a phylogenic tree of the 51 species included in our dataset from the phylogenetic super-tree of mammals published by Bininda-Emonds et al. [54], which provides information on both topology and branch length. We then performed the analysis by using Phylogenetic Generalized Least-Squares models (PGLS), a statistical method that provides an estimate of the phylogenetic correlation (named ‘λ’) and allows phylogenetic dependence to be controlled [55]. After having checked data normality using Shapiro-Wilk tests and applied log-transformations when necessary, we ran a series of models including aging rate (log-transformed absolute value) or median lifespan (log-transformed) as dependent variables and testes mass (log-transformed), age at first reproduction (log-transformed) and their possible two-way interaction (assessed by the product of these two continuous variables) as independent variables. As it is strongly advocated to avoid the use of residuals as independent variables [56], [57], we measured species relative allocation to testes mass by including male body mass (log-transformed) as a covariate in all models that also included testes mass (log-transformed) (e.g. [58], [59], [60]). Models were first run on the total number of species (*N* = 51) and then separately on Artiodactyla and Carnivora because of the large sample size available in these groups (*N* = 20 and 15, respectively). Finally, on the subset of 17 species with information on multipaternities, we fitted a series of models including female aging rate (absolute values) or median lifespan as dependent variables and proportion of multiple paternities, litter size, age at first reproduction and two-way interaction terms as independent variables. All variables were log-transformed. We included litter size as a covariate in these models because the likelihood of detecting multiple sired litters increases with the species litter size [41].We selected the best models based on the Akaike information criterion corrected for small sample size (AICc) and we calculated AICc weights (w_i_) to assess the relative likelihood of each model to be the best among all the fitted models [61]. We selected the model with the lowest AICc. When the difference of AICc between two competing models was within 2 units, we retained the simplest model to satisfy parsimony rules [61]. We used R version 2.12.1 (R Development Core Team 2011) to perform our analysis. Results are presented as mean ± SEM.

## Results

### Median Lifespan

**Figure 1:**
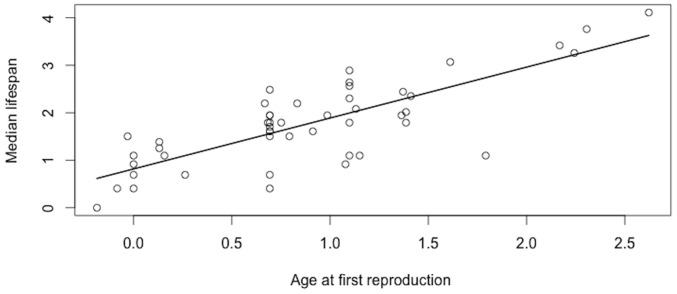
Relationship between median lifespan and age at first reproduction (on a log-scale) of females across 48 mammalian species.

**Figure 2:**
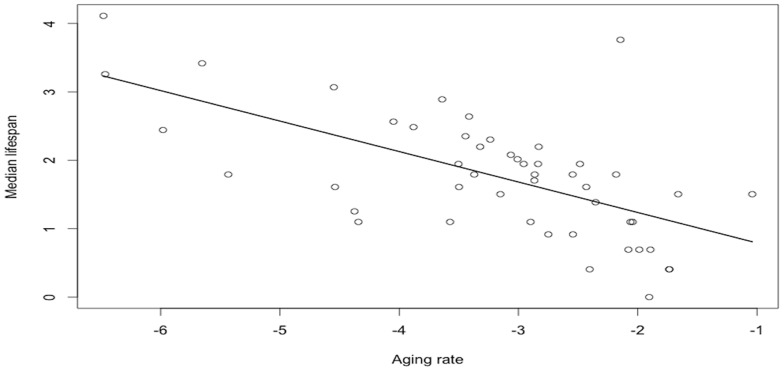
Relationship between median lifespan and aging rate (on a log-scale) of females across 48 mammalian species.

The best model explaining variation in female median lifespan included only age at first reproduction (w_i = _0.88; Table 2). As expected, female median lifespan increased with age at first reproduction (β = 1.02±0.14; *t* = 7.08; P<0.0001; Figure 1). The same model was the best also in the subset containing Artiodactyla only (β = 1.20±0.28; *t* = 4.26; P<0.001), while in Carnivora, the constant model performed similarly to the model including the age at first reproduction (w_i_ = 0.43 and 0.41 respectively) and was retained. In either case, we did not find any evidence for a relationship between the relative testes mass and median lifespan, when either including the effect of the age at first reproduction or not (Table 2 and 3). Estimated effect sizes were relatively small and of variable sign (β = 0.05±0.09, −0.16±0.13, and 0.18±0.23 for all groups, Artiodactyla, and Carnivora, respectively), indicating random rather than structured variation. Moreover, adding testes mass to these models does not improve the fit of the data (see R^2^, Table 2). All models including the proportion of multiple paternities performed poorly compared to the constant model (Table 4). In particular, we did not find any evidence for a relationship between the proportion of multiple paternities and median lifespan (β = 0.11±0.18, Table 4). Finally, as expected female median lifespan was negatively correlated with aging rate across mammalian species (β = −0.32±0.08; *t* = −3.97; P<0.001; Figure 2).

**Table 2:** Analysis of the influence of age at first reproduction and relative testes mass on female median lifespan and aging rate.

			Median lifespan						Aging rate					
	Independent variables	k	λ	R^2^	Deviance	AICc	ΔAICc	w_i_	λ	R^2^	Deviance	AICc	ΔAICc	w_i_
All species(n = 48 and 51)	Constant	3	0.86	0.00	103.22	105.32	27.97	0.00	0.50	0.00	174.68	176.77	10.98	0.00
	Age at first reproduction	4	0.57	0.51	72.04	77.35	0.00	0.88*	0.08	0.31	160.5	165.79	0.00	0.82*
	Body mass	4	0.63	0.34	85.48	90.78	13.43	0.00	0.38	0.05	171.34	176.64	10.85	0.00
	Testes mass+Body mass	5	0.63	0.34	84.50	93.15	15.80	0.00	0.38	0.03	171.34	179.95	14.16	0.00
	Age at first reproduction+Testes mass+Body mass	6	0.48	0.52	66.76	82.88	5.53	0.06	<0.001	0.37	157.38	169.43	3.64	0.13
	Age at first reproduction*Testes mass+Body mass	7	0.48	0.55	67.08	82.78	5.43	0.06	<0.001	0.37	156.32	171.91	6.12	0.04
All species(n = 50)	Constant	3							0.67	0.00	156.42	158.51	13.41	0.00
	Age at first reproduction	4							<0.001	0.39	139.8	145.10	0.00	0.87*
	Body mass	4							0.44	0.06	153.32	158.61	13.51	0.00
	Testes mass+Body mass	5							0.43	0.04	153.3	161.91	16.81	0.00
	Age at first reproduction+Testes mass+Body mass	6							<0.001	0.39	137.28	149.34	4.24	0.10
	Age at first reproduction*Testes mass+Body mass	7							<0.001	0.38	136.74	152.37	7.27	0.02
Carnivores(n = 14 and 15)	Constant	3	<0.001	0.00	17.90	20.27	0.00	0.43*	0.79	0.00	44.26	46.61	0.00	0.66*
	Age at first reproduction	4	<0.001	0.17	14.12	20.36	0.09	0.41	0.30	0.11	43.24	49.39	2.78	0.16
	Body mass	4	<0.001	0.03	16.36	22.61	2.34	0.13	0.80	0.00	44.26	50.41	3.80	0.10
	Testes mass+Body mass	5	<0.001	0.00	16.00	26.79	6.52	0.02	0.81	0.09	40.54	51.08	4.47	0.07
	Age at first reproduction+Testes mass+Body mass	6	<0.001	0.09	12.84	28.99	8.72	0.01	0.40	0.13	39.7	55.38	8.77	0.01
	Age at first reproduction*Testes mass+Body mass	7	<0.001	0.17	10.18	32.87	12.60	0.00	<0.001	0.31	38.18	59.92	13.31	0.00
Artiodactyles(n = 19)	Constant	3	0.65	0.00	29.86	32.12	9.54	0.01	<0.001	0.00	38.64	40.90	0.00	0.60*
	Age at first reproduction	4	0.83	0.49	16.72	22.58	0.00	0.73*	<0.001	0.04	36.76	42.63	1.73	0.25
	Body mass	4	0.75	0.14	26.04	31.91	9.33	0.01	<0.001	0.00	38.5	44.35	3.45	0.11
	Testes mass+Body mass	5	0.74	0.16	24.32	34.19	11.61	0.00	<0.001	0.00	37.16	47.03	6.13	0.03
	Age at first reproduction+Testes mass+Body mass	6	0.87	0.52	13.42	24.77	2.19	0.24	<0.001	0.00	35.52	49.86	8.96	0.01
	Age at first reproduction*Testes mass+Body mass	7	0.88	0.53	12.10	31.50	8.92	0.01	<0.001	0.00	34.48	53.89	12.99	0.00

We compared models based on AICc and w_i_ (see Material and methods section). K represents the number of parameters in the model, R^2^ corresponds to the adjusted R^2^ and Δ AICc represents the difference of corrected Akaike’s with that of the best models (*).

1 For the moose, *Alces alces*, the body mass extracted from the testes mass source was really high (789 kg, [81]). However, results were unchanged when we used body mass from another source (323 Kg, [82]).

**Table 3:** Parameters of the model testing the relationship between testes mass (controlled for body mass) and both median lifespan and aging rate.

		Median lifespan		Aging rate	
		Estimate± SE	*t*	Estimate± SE	*t*
All species(*N* = 51)	Body mass	0.18±0.08	2.15	−0.17±0.18	−0.93
	Testes mass	0.10±0.10	0.95	−0.01±0.23	−0.05
All species(*N* = 50)	Body mass			−0.19±0.16	−1.20
	Testes mass			0.03±0.20	0.16
Carnivores(*N* = 14)	Body mass	−0.01±0.19	−0.07	−0.79±0.47	−1.69
	Testes mass	0.13±0.24	0.52	1.09±0.59	1.84
Artiodactyles(*N* = 19)	Body mass	0.60±0.13	2.3	0.25±0.23	1.07
	Testes mass	−0.20±0.16	−1.23	−0.14±0.20	−0.70

All variables are log-transformed and effects size are reported accordingly.

**Table 4:** Analysis of the influence of age at first reproduction, litter size (LS) and proportion of multiple paternities (MP) on female median lifespan and aging rate.

			Median lifespan					Aging rate					
Independant variables	k	λ	R^2^	Deviance	AICc	ΔAICc	w_i_	λ	R^2^	Deviance	AICc	ΔAICc	w_i_
Constant	3	<0.001	0.00	24.82	27.15	0.00	0.51*	0.78	0.00	46.56	48.88	0.00	0.42
Age at first reproduction	4	<0.001	0.00	23.82	29.89	2.74	0.13	0.73	0.00	46.56	52.61	3.73	0.07
MP	4	<0.001	0.00	24.38	30.44	3.29	0.10	0.81	0.00	45.92	51.96	3.08	0.09
LS	4	<0.001	0.05	22.86	28.91	1.76	0.21	<0.001	0.22	43.34	49.39	0.51	0.33*
MP+LS	5	<0.001	0.00	22.84	33.17	6.02	0.02	<0.001	0.16	43.32	53.63	4.75	0.04
MP*LS	5	<0.001	0.00	22.52	37.75	10.60	0.00	<0.001	0.10	43.16	58.40	9.52	0.00
Age at first reproduction+MP	5	<0.001	0.00	22.88	33.21	6.06	0.02	<0.001	0.14	43.68	54.00	5.12	0.03
Age at first reproduction+MP+LS	6	<0.001	0.00	21.78	37.02	9.87	0.00	<0.001	0.24	40.34	55.58	6.70	0.01
Age at first reproduction+MP×LS	7	<0.001	0.00	21.20	42.19	15.04	0.00	<0.001	0.18	40.32	61.31	12.43	0.00

We compared models based on AICc and w_i_ (see Material and methods section). K represents the number of parameters in the model, R^2^ corresponds to the adjusted R^2^ and Δ AICc represents the difference of corrected Akaike’s with that of the best models (*).

### Aging Rate

**Figure 3:**
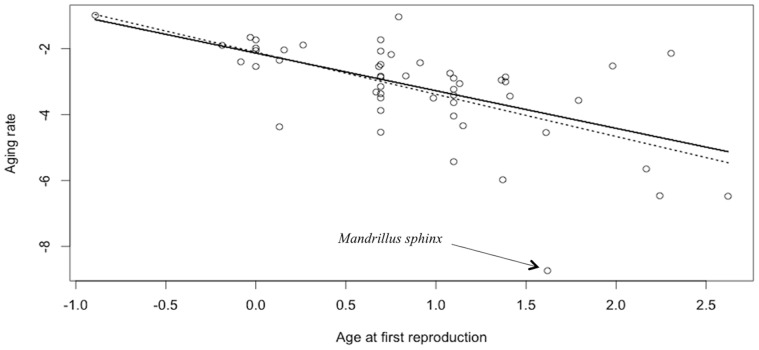
Relationship between aging rate and age at first reproduction of females across mammalian species. The dash line represents the relationship when *Mandrillus sphinx* is removed due to its extremely low aging rate.

All species showed the expected increase in mortality rate with age (see Table S1 and S2). The best model of variation in aging rate only included the variable age at first reproduction (w_i_ = 0.82; Table 2). As expected the rate of aging decreased with increasing age at first reproduction (β = −1.21±0.25; *t* = −4.86; P<0.001). One species, *Mandrillus sphinx,* had a very low aging rate compared to other species (Figure 3, see also Table S1); but excluding *M. sphinx* did not qualitatively affect the results (Table 2). In particular the relationship between aging rate and age at first reproduction remained the same (β = −1.14±0.20; *t* = −5.65; P<0.001; Figure 3). Moreover and similarly to our observations for median lifespan, estimated effect sizes for testes mass were also relatively small and of variable sign (Table 3). Additionaly, R^2^ of models including testes mass were particularly low (Table 2). When looking within Carnivora or Artiodactyla separately, the effect of age at first reproduction vanished. In both groups the constant model was retained (Table 2). In either case, we did not find any evidence for a relationship between relative testes mass and aging rate, when either including the effect of the age at first reproduction or not (Table 2 and 3). Finally, we did not find any effect of the proportion of multiple paternity on aging rate (β = −0.25±0.32, Table 4). The best model only included litter size (w_i_ = 0.33; Table 4) with the expected positive effect (*n* = 16; β = 1.25±0.55; *t* = 2.28; P<0.038) but based on parsimony rules, we retained the constant model (ΔAICc between these two models = 0.51; Table 4).

## Discussion

Our results do not support the hypothesis that multiple mating with different males increases female mortality in mammals. Indeed none of our measures of polyandry were correlated with female median lifespan or aging rate. Although relationships at the inter-specific level do not always reflect relationships at the intra-specific level, our results obtained across several mammalian species contrast with several studies in invertebrates where polyandrous females were found to have a lower lifespan than monandrous females ([9], [62], but see [63]).

Some case studies have reported that polyandrous females can show increased lifespan, essentially through nuptial gifts provided by males [4], [62]. To date these examples remain limited to intra-specific studies in invertebrates like southern stink bugs (*Nezara viridula*) in which females that mate repeatedly live three or more times longer than females that mate only once or twice [64]. Although we did not detect any positive relationship between polyandry and female mortality across our sample of mammalian species, it is possible that polyandry reduces female survival in some species and increases it in others, which could potentially explain our results.

In mammals, the richness and prevalence of sexually-transmitted diseases is high [11] and the complex copulatory behaviours such as prolonged intromissions in Rodentia [65], copulatory lock in Carnivora, or multiple ejaculations in Artiodacyla or Primates [66] require a high degree of physical contacts between males and females, which is likely to increase the risk of contracting a sexually-transmitted disease. Therefore, females from polyandrous species might have developed counter-adaptations to limit this risk. For example, in primates, females from polyandrous species have a higher level of lymphocytes and monocytes [67], [68] and some of the genes involved in the immune system evolve at a faster rate compared to monandrous females [69]. Additionally, it has been suggested that some genital grooming behaviour might have evolved in females to reduce the likelihood of contracting pathogens [70] although evidence for this remains equivocal in primates [71]. Overall, these counter-adaptations in females might reduce the mortality costs of polyandry in mammals.

The sharp penile spines sometimes present in mammalian genitalia [72] could potentially lead to lethal injuries making polyandrous females more exposed to death risk following mating. However, such situations appear unlikely since reproductive success in males is strongly dependent on maternal care to offspring and thus on female survival [73]. Moreover, results from a comparative inter-specific study in Primates suggest that penile spines may decrease rather than increase the risk of lethal injuries during a reproductive cycle since in Primates, the presence of penile spines may reduce female receptivity and thereby the potential for multiple mating [74]. Finally, it has been suggested that polyandry could also have evolved to reduce costs associated with male harassment [4], [75]. For instance, in South American sea lions (*Otaria flavescens*) females increase their breeding group size to reduce the level of male sexual harassment [76], which ultimately should reduce the associated mortality costs. Although reports of harassment or coercive mating in species included in our analysis remain limited (but see [77] for a good evidence of female mortality due to male harassment in feral sheep, *Ovis aries*), this hypothesis might to some extent explain our results.

Our study also emphasizes the importance of controlling for the species pace of life in interspecific analyses testing hypotheses on the evolution of life-history traits such as lifespan or aging rate [49]. Indeed, as expected, we found a close association between age at first reproduction and both median lifespan and aging rate across mammal species. This adds to the compelling evidence that mammalian species can be ranked along a slow-fast continuum where fast species reproduce early, have a high allocation to reproduction early in life, a short longevity and a steep rate of aging. On the contrary slow species first reproduce late, have a long life and a low rate of aging [46], [49], [51].

To conclude, we found no evidence that polyandry induces mortality costs in female mammals. However, we emphasize that our study is only a first step in the understanding of costs of polyandry in this group. Indeed high quality data from long-term longitudinal studies are rapidly accumulating [78]. This will hopefully allow our analyses to be refined by including other key-aspects that could influence the potential relationships between polyandry, lifespan and aging, like an index of penile spinosity, the size of seminal vesicles (in mammalian taxa where they occur), or the diversity in sexually transmitted diseases. Moreover, refined long-term datasets will also allow any relationship between polyandry and reproductive senescence to be detected, as multiple mating has previously been shown to cause a stronger reproductive decline in invertebrates [62]. In the meantime, we suggest that an experimental approach with controlled manipulation of female multiple mating associated with anatomical and behavioural measures of females could help uncover the relationship between male mating behaviour, complex reproductive anatomies and mortality risk in mammals. Overall, information from experimental, comparative and meta-analytical approaches will markedly improve our understanding of the relationships between sex-specific allocation to sexually selected traits and aging patterns [79], [80].

## Supporting Information

Table S1 List of all species (*N* = 51) and median lifespan/aging rate data included in the analysis.
(DOC)

Table S2 List of all species (*N* = 51) and data on reproductive traits included in the analysis.
(DOC)

Table S3 References for table S1 and S2.
(DOC)
