# Supplementary material for: Polyandry Has No Detectable Mortality Cost in Female Mammals
Source: PLoS One. 2013 Jun 18;8(6):e66670. doi: 10.1371/journal.pone.0066670 (PMC3688942; doi:10.1371/journal.pone.0066670)
Supplement: Table S1 — List of all species (N = 51) and median lifespan/aging rate data included in the analysis. (DOC) [file pone.0066670.s001.doc]

**SUPPORTING INFORMATION**

**Polyandry has no detectable mortality cost in female mammals**

Jean-François Lemaître and Jean-Michel Gaillard

**Table S1:** List of all species (*N* = 51) and median lifespan / aging rate data included in the analysis. * species where age-specific information was extracted from graphs.

| **Common name** | **Latin name** | **Median lifespan** | **Aging rate** | **References** |
| --- | --- | --- | --- | --- |
| Impala | *Aepyceros melampus* | 6 | -0.078 | Spinage 1972 |
| Moose | *Alces alces* | 7 | -0.083 | Ericsson and Wallin 2001 |
| Pronghorn | *Antilocapra americana* | 12 | -0.021 | Byers 1997 |
| Alpine ibex | *Capra ibex* | 13 | -0.017 | Toïgo 2007 |
| Iberian ibex | *Capra pyrenaica* | 6 | -0.034 | Chirosa et al. 2001 |
| Roe deer | *Capreolus capreolus* | 5.5 | -0.057 | Gaillard et al. 2004 |
| Elk | *Cervus canadensis* | 18 | -0.02 | Houston 1982 |
| Red deer | *Cervus elaphus* | 10 | -0.039 | Catchpole et al. 2004* |
| Blue wildebeeste | *Connochaetes taurinus* | 5 | -0.088 | Attwell 1982* |
| Himalayan Tahr | *Hemitragus jemlahicus* | 7 | -0.059 | Caughley 1966 |
| Defassa Waterbuck | *Kobus ellipsiprymnus* | 7 | -0.030 | Spinage 1970 |
| Lechwe | *Kobus leche* | 6 | -0.113 | Sayer and VanLavieren 1975 |
| Black-tailed deer | *Odocoileus hemionus* | 5 | -0.011 | Taber and Dasmann 1957 |
| White-tailed deer | *Odocoileus virginianus* | 4.5 | -0.043 | DelGiudice et al. 2006 |
| Soay sheep | *Ovis aries* | 2.5 | -0.079 | Catchpole et al. 1988* |
| Bighorn sheep | *Ovis canadensis* | 8 | -0.047 | Loison et al. 1999 |
| Reindeer | *Rangifer tarandus* | 9 | -0.059 | Reimers 1983; Leader-Williams 1988 |
| Wild Boar | *Sus scrofa* | 1.5 | -0.176 | Schauss 1990 |
| Greater kudu | *Tragelaphus strepsiceros* | 9 | -0.036 | Owen-Smith 1990 |
| Northern Fur Seal | *Callorhinus ursinus* | 7.5 | -0.049 | Lander 1981 |
| Spotted hyena | *Crocuta crocuta* | 3 | -0.055 | Frank et al. 1995* |
| Dwarf mangoose | *Helogale parvula* | 3.5 | -0.012 | Waser et al. 1995 |
| European Otter | *Lutra lutra* | 5 | -0.030 | Kruuk et al. 1987* |
| African wild dog | *Lycaon pictus* | 4.5 | -0.353 | Creel & Creel 2002 |
| Badger | *Meles meles* | 4 | -0.095 | Wilkinson et al. 2000 |
| Striped skunk | *Mephitis mephitis* | 3 | -0.130 | Casey and Webster 1975* |
| American mink | *Mustela vison* |  | -0.371 | Bonesi et al. 2006* |
| Raccoon dog | *Nyctereutes procyonoides* | 1.5 | -0.090 | Helle and Kauhala 1993 |
| Lion | *Panthera leo* | 6 | -0.057 | Packer et al. 1988* |
| Gray Fox | *Urocyon cinereoargenteus* | 1.5 | -0.176 | Wigal and Chapman 1983 |
| Black bear | *Ursus americanus* | 6 | -0.004 | Koehler and Pierce 2005* |
| Brown Bear | *Ursus arctos* | 3 | -0.028 | Knight and Eberhardt 1985 |
| Polar bear | *Ursus maritimus* | 3 | -0.013 | Regher et al. 2007 |
| Red Fox | *Vulpes vulpes* | 4.5 | -0.190 | Soulsbury 2008* |
| European hedgehog | *Erinaceus europaeus* | 2 | -0.137 | Kristiansson 1990 |
| Grant’s Zebra | *Equus burchellii* | 10.5 | -0.032 | Spinage 1972 |
| Feral horse | *Equus caballus* | 14 | -0.032 | Garrott and Taylor 1990 |
| Indian rhinoceros | *Rhinoceros unicornis* |  | -0.080 | Dinerstein and Price 1991 |
| Patas monkey | *Erythrocebus patas* | 2.5 | -0.064 | Nakagawa et al. 2003 |
| Easter gorilla | *Gorilla gorilla* | 30.5 | -0.003 | Bronikowski et al. 2011* |
| Japanese macaques | *Macaca fuscata* | 21.5 | -0.010 | Fedigan and Zohar 1997 |
| Pig-tailed macaques | *Macaca nemestrina* | 11.5 | -0.002 | Nelson & Chapman 1982 |
| Mandrill | *Mandrillus sphinx* |  | -0.0001 | Setchell et al. 2005* |
| Chimpanzee | *Pan troglodytes* | 26 | -0.001 | Bronikowski et al. 2011* |
| Gelada baboon | *Theropithecus gelada* | 7 | -0.052 | Dunbar 1980 |
| Asian elephant | *Elephas maximus* | 61 | -0.001 | Sukumar et al. 1997 |
| African elephant | *Loxodonta africana* | 43 | -0.117 | Moss 2001* |
| Black-tailed prairie dog | *Cynomys ludovicianus* | 2 | -0.125 | Hoogland 1995 |
| Golden-mantled ground squirrel | *Spermophilus lateralis* | 2 | -0.150 | Bronson 1979 |
| American red squirrel | *Tamiasciurus hudsonicus* | 3 | -0.127 | Descamps et al. 2008 |
| Eastern mole | *Scalopus aquaticus* | 1 | -0.149 | Davis and Choate 1993 |
