# Supplementary material for: Polyandry Has No Detectable Mortality Cost in Female Mammals
Source: PLoS One. 2013 Jun 18;8(6):e66670. doi: 10.1371/journal.pone.0066670 (PMC3688942; doi:10.1371/journal.pone.0066670)
Supplement: Table S2 — List of all species (N = 51) and data on reproductive traits included in the analysis. (DOC) [file pone.0066670.s002.doc]

**SUPPORTING INFORMATION**

**Polyandry has no detectable mortality cost in female mammals**

Jean-François Lemaître and Jean-Michel Gaillard

**Table S2:** List of all species (*N* = 51) and data on reproductive traits included in the analysis.

| **Order** | **Common name** | **Latin name** | **Testes mass (g)** | **Male body mass (g)** | **References** | **Age at first reproduction (years)** | **References** | **Litter size** | **Multiple paternities (%)** | **References** |
| --- | --- | --- | --- | --- | --- | --- | --- | --- | --- | --- |
| Artiodactyla | Impala | *Aepyceros melampus* | 120 | 62500 | Ginsberg and Rubenstein 1990 | 1.98 | Wootton 1987 |  |  |  |
| Artiodactyla | Moose | *Alces alces* | 106 | 789000 | Ginsberg and Rubenstein 1990; Silva and Downing | 2 | Wootton 1987 |  |  |  |
| Artiodactyla | Pronghorn | *Antilocapra americana* | 76 | 61700 | Ginsberg and Rubenstein 1990 | 2 | Wootton 1987 | 1.93 | 44.00 | Jones et al. 2009; Soulsbury 2010 |
| Artiodactyla | Alpine ibex | *Capra ibex* | 38.34 | 75000 | Anderson et al. 2004 | 3 | Wootton 1987 |  |  |  |
| Artiodactyla | Iberian ibex | *Capra pyrenaica* | 39.64 | 39314.57 | Sarasa et al. 2010 | 2 | Alados & Escos 1988;  Grzimek 1990 |  |  |  |
| Artiodactyla | Roe deer | *Capreolus capreolus* | 45 | 20400 | Kenagy and Trombulak 1986 | 2 | Wootton 1987 | 1.76 | 15.5 | Soulsbury 2010 |
| Artiodactyla | Elk | *Cervus canadensis* | 220 | 280000 | Ginsberg and Rubenstein 1990 | 3 | Gaillard et al. 1989 |  |  |  |
| Artiodactyla | Red deer | *Cervus elaphus* | 218 | 122000 | Ginsberg and Rubenstein 1990 | 3 | Gaillard et al. 1989 |  |  |  |
| Artiodactyla | Blue wildebeeste | *Connochaetes taurinus* | 306 | 227000 | Ginsberg and Rubenstein 1990 | 2.49 | Wootton 1987 |  |  |  |
| Artiodactyla | Himalayan Tahr | *Hemitragus jemlahicus* | 26.7 | 76000 | Anderson et al. 2004 | 2 | Caughley 1966 |  |  |  |
| Artiodactyla | Defassa Waterbuck | *Kobus ellipsiprymnus* | 148 | 243200 | Ginsberg and Rubenstein 1990 | 2.68 | Wootton 1987 |  |  |  |
| Artiodactyla | Lechwe | *Kobus leche* | 67.85 | 109000 | Tourmente et al. 2011 | 2.12 | Wootton 1987 |  |  |  |
| Artiodactyla | Black-tailed deer | *Odocoileus hemionus* | 87 | 112000 | Ginsberg and Rubenstein 1990 | 2 | Mueller & Sadleir 1979 |  |  |  |
| Artiodactyla | White-tailed deer | *Odocoileus virginianus* | 76 | 71000 | Ginsberg and Rubenstein 1990 | 2 | Wootton 1987 | 2.00 | 22.2 | Soulsbury 2010 |
| Artiodactyla | Soay sheep | *Ovis aries* | 318.8 | 35000 | Soulsbury 2010 | 1 | Clutton-Brock & Pemberton 2004 | 2.00 | 73.8 | Soulsbury 2010 |
| Artiodactyla | Bighorn sheep | *Ovis canadensis* | 338 | 122000 | Ginsberg and Rubenstein 1990 | 3.1 | Wootton 1987 |  |  |  |
| Artiodactyla | Reindeer | *Rangifer tarandus* | 132 | 145000 | Ginsberg and Rubenstein 1990 | 2.3 | Wootton 1987 |  |  |  |
| Artiodactyla | Wild Boar | *Sus scrofa* | 128.2 | 39700 | Tourmente et al. 2011 | 2 | Heise-Pavlov et al. 2009 | 5.39 | 32.53 | Soulsbury 2010 |
| Artiodactyla | Greater kudu | *Tragelaphus strepsiceros* | 92 | 248000 | Ginsberg and Rubenstein 1990 | 1.95 | Wootton 1987 |  |  |  |
| Carnivora | Northern Fur Seal | *Callorhinus ursinus* | 63.6 | 29000 | Kenagy and Trombulak 1986 | 4 | Wootton 1987 |  |  |  |
| Carnivora | Spotted hyena | *Crocuta crocuta* | 9.85 | 45000 | Iossa et al. 2008 | 3 | Frank et al. 1995 | 1.51 | 26.55 | Soulsbury 2010 |
| Carnivora | Dwarf mangoose | *Helogale parvula* | 0.45 | 455 | Anderson et al. 2004 | 1.14 | Wootton 1987 | 2.83 | 19,00 | Soulsbury 2010 |
| Carnivora | European Otter | *Lutra lutra* | 4.05 | 7825 | Tourmente et al. 2011 | 2 | Hamilton & Eadie 1964 |  |  |  |
| Carnivora | African wild dog | *Lycaon pictus* | 28.6 | 31464.29 | Tourmente et al. 2011 | 2.21 | Wootton 1987 | 8.1 | 10,00 | Jones et al. 2009; Soulsbury 2010 |
| Carnivora | Badger | *Meles meles* | 14.4 | 14515 | Kenagy and Trombulak 1986 | 1.14 | Wootton 1987 | 1.43 | 30.5 | Soulsbury 2010 |
| Carnivora | Striped skunk | *Mephitis mephitis* | 5.05 | 5050 | Iossa et al. 2008 | 1.17 | Wootton 1987 |  |  |  |
| Carnivora | American mink | *Mustela vison* | 5.4 | 2387.6 | Tourmente et al. 2011 | 0.41 | Wootton 1987 | 3.16 | 33.33 | Soulsbury 2010 |
| Carnivora | Raccoon dog | *Nyctereutes procyonoides* | 5.64 | 4135 | Tourmente et al. 2011 | 0.92 | Wootton 1987 |  |  |  |
| Carnivora | Lion | *Panthera leo* | 55 | 188000 | Iossa et al. 2008 | 4 | Wootton 1987 | 2.75 | 14.2 | Soulsbury 2010 |
| Carnivora | Gray Fox | *Urocyon cinereoargenteus* | 5.07 | 3719 | Iossa et al. 2008 | 1 | Wootton 1987 | 3.3 | 14.3 | Soulsbury 2010 |
| Carnivora | Black bear | *Ursus americanus* | 38.9 | 102287 | Soulsbury 2010 | 3 | Wootton 1987 | 2.4 | 35.5 | Soulsbury 2010 |
| Carnivora | Brown Bear | *Ursus arctos* | 68.6 | 180900 | Tourmente et al. 2011 | 6 | Wootton 1987 | 2,00 | 20.7 | Soulsbury 2010 |
| Carnivora | Polar bear | *Ursus maritimus* | 131.4 | 427000 | Iossa et al. 2008 | 3.16 | Wootton 1987 |  |  |  |
| Carnivora | Red Fox | *Vulpes vulpes* | 9 | 5069 | Kenagy and Trombulak 1986 | 0.97 | Wootton 1987 | 3.2 | 18.75 | Soulsbury 2010 |
| Erinaceomorpha | European hedgehog | *Erinaceus europaeus* | 2.31 | 665 | Kenagy and Trombulak 1986 | 1 | Gaillard et al. 1989 |  |  |  |
| Perissodactyla | Grant’s Zebra | *Equus burchellii* | 302 | 238000 | Ginsberg and Rubenstein 1990 | 4.1 | Wootton 1987 |  |  |  |
| Perissodactyla | Feral horse | *Equus caballus* | 416 | 450000 | Ginsberg and Rubenstein 1990 | 3 | Garrott & Taylor 1990 |  |  |  |
| Perissodactyla | Indian rhinoceros | *Rhinoceros unicornis* | 2600 | 2000000 | Endo et al. 1996 | 7.25 | Dinerstein and Price 1991 |  |  |  |
| Primates | Patas monkey | *Erythrocebus patas* | 7.2 | 13000 | Tourmente et al. 2011 | 2.94 | Wootton 1987 |  |  |  |
| Primates | Easter gorilla | *Gorilla gorilla* | 23.2 | 134000 | Kenagy and Trombulak 1986 | 8.74 | Wootton 1987 |  |  |  |
| Primates | Japanese macaques | *Macaca fuscata* | 72.3 | 15400 | Dixson and Anderson 2004 | 5.01 | Wootton 1987 |  |  |  |
| Primates | Pig-tailed macaques | *Macaca nemestrina* | 66.7 | 9980 | Kenagy and Trombulak 1986 | 3.94 | Wootton 1987 |  |  |  |
| Primates | Mandrill | *Mandrillus sphinx* | 58.86 | 26900 | Dixson and Anderson 2004 | 5.05 | Wootton 1987 |  |  |  |
| Primates | Chimpanzee | *Pan troglodytes* | 118.8 | 44340 | Kenagy and Trombulak 1986 | 9.42 | Wootton 1987 |  |  |  |
| Primates | Gelada baboon | *Theropithecus gelada* | 21.5 | 20400 | Tourmente et al. 2011 | 3.91 | Wootton 1987 |  |  |  |
| Proboscidea | Asian elephant | *Elephas maximus* | 4000 | 4545000 | Ginsberg and Rubenstein 1990 | 13.76 | Wootton 1987 |  |  |  |
| Proboscidea | African elephant | *Loxodonta africana* | 4530 | 4365000 | Ginsberg and Rubenstein 1990 | 10.03 | Wootton 1987 |  |  |  |
| Rodentia | Black-tailed prairie dog | *Cynomys ludovicianus* | 4 | 815 | Ramm et al. 2005 | 2 | Wootton 1987 | 4.43 | 2.9 | Jones et al. 2009; Hoogland and Foltz 1982 |
| Rodentia | Golden-mantled ground squirrel | *Spermophilus lateralis* | 3.25 | 187.5 | Kenagy and Trombulak 1986 | 1.3 | Wootton 1987 |  |  |  |
| Rodentia | American red squirrel | *Tamiasciurus hudsonicus* | 2.92 | 186 | Soulsbury 2010 | 1 | Wootton 1987 | 3.00 | 77.15 | Soulsbury 2010 |
| Soricomorpha | Eastern mole | *Scalopus aquaticus* | 2.2 | 125.1 | Kenagy and Trombulak 1986 | 0.83 | Wootton 1987 |  |  |  |
