# Supplementary material for: Polyandry Has No Detectable Mortality Cost in Female Mammals
Source: PLoS One. 2013 Jun 18;8(6):e66670. doi: 10.1371/journal.pone.0066670 (PMC3688942; doi:10.1371/journal.pone.0066670)
Supplement: Table S3 — References for table S1 and S2. (DOC) [file pone.0066670.s003.doc]

**SUPPORTING INFORMATION**

**Polyandry has no detectable mortality cost in female mammals**

Jean-François Lemaître and Jean-Michel Gaillard

**Table S3**: References for Table S1 and S2

Alados CL, Escós J (1988) Parturition dates and mother-kid behavior in Spanish ibex (*Capra pyrenaica*) in Spain. J Mammal 69: 172-175.

Anderson MJ, Nyholt J, Dixson AF (2004) Sperm competition affects the structure of the mammalian vas deferens. J Zool 264: 97-103.

Attwell CAM (1982) Population ecology of the wildebeest *Connochaetes taurinus taurinus* in Zululand, South Africa. Afr J Ecol 20: 147-168.

Bonesi L, Harrington LA, Maran T, Sidorovich VE, McDonald DW (2006) Demography of the populations of American mink *Mustela vison* in Europe. Mammal Rev 36: 98-106.

Bronikowski AM, Altmann J, Brockman DK, Cords M, Fedigan LM et al. 2011. Aging in the natural world: Comparative data reveal similar mortality patterns across primates. Science 331: 1325–1328.

Bronson MT (1979) Altitudinal variation in the life history of the golden-mantled ground squirrel (*Spermophilus lateralis*). Ecology 60: 272–279.

Byers J (1997) American Pronghorn: Social Adaptations and the Ghosts of Predators Past. Chicago University Press, Chicago.

Casey GA, Webster WA (1975) Age, sex determination of skunk (*Mephitis mephitis*) from Ontario, Manitoba and Quebec. Can J Zool 53: 223-231.

Catchpole EA, Morgan BJT, Freeman SN, Albon SD, Coulson TN (1998) An integrated analysis of Soay sheep survival data. University of Kent.

Catchpole EA, Fan Y, Morgan BJT, Clutton-Brock TH, Coulson T (2004) Sexual dimorphism, survival and dispersal in red deer. JABES 9: 1-26.

Caughley G (1966) Mortality patterns in mammals. Ecology 47: 906–918.

Chirosa M, Delibes JR, Fandos P, Granados JE, Pérez MC, Pérez JM, Ruiz-Martinez I, Serrano E, Soriguer RC (2001) Estimas poblacionales de *Capra pyrenaica hispanica*. Unpublished report. Universidad de Jaén, Spain.

Clutton-Brock TH, Pemberton J (2004) Soay sheep: dynamics and selection in an island population. Cambridge, UK: Cambridge University Press.

Creel S, Creel NM (2002) The African wild dog: Behavior, ecology, and conservation. Princeton: Princeton University Press.

Davis FW, Choate JR (1993) Morphologic variation and age structure in a population of the eastern mole, *Scalopus aquaticus*. J Mammal 74: 1014-1025.

DelGiudice GD, Fieberg J, Riggs MR, Carstensen Powell M, Pan W (2006) A long-term age-specific survival analysis of female white-tailed deer. J Wild Manag 70: 1556-1568.

Descamps S, Boutin S, Berteaux D, Gaillard JM (2008) Age-specific variation in survival, reproductive success and offspring quality in red squirrels: evidence of senescence. Oikos 117: 1406-1416.

Dinerstein E, Price CL (1991) Demography and habitat use by Greater One Horned Rhinoceros in Nepal. J Wildl Manage 55: 401-411.

Dixson AF, Anderson MJ (2004) Sexual behavior, reproductive physiology and sperm competition in male mammals. Physiol Behav 83: 361-371.

Dunbar RIM (1980) Demographic and life history variables of a population of gelada babbons (*Theropithecus gelada*). J Anim Ecol 49: 485-506.

Endo H, Yamada TK, Nakamuta N, Tanemura K, Kurohmaru M, Hayashi Y (1996) Testicular morphology of a greater Indian Rhinoceros (*Rhinoceros unicornis*). J Vet Med Sci 58: 937-940.

Ericsson G, Wallin K (2001) Age-specific moose (*Alces alces*) mortality in a predator-free environment: Evidence for senescence in females. Ecoscience 8: 157-163.

Fedigan LM, Zohar S (1997) Sex differences in mortality of Japanese Macaques: Twenty-one years of data from the Arashiyama West Population. Am J Phys Anthropol 102: 161-175.

Frank LG, Holekamp KE, Smale E (1995) Dominance, Demography, and Reproductive Success of Female Spotted Hyenas. In: Serengeti II, Dynamics, Management, and Conservation of an Ecosystem (pp. 364-384). Eds. Sinclair and Arcese. The University of Chicago Press, Chicago and London.

Gaillard JM, Pontier D, Allaine D, Lebreton J, Trouvilliez J, Clobert J (1989) An analysis of demographic tactics in birds and mammals. Oikos 56: 59-76.

Gaillard JM, Viallefont A, Loison A, Festa-Bianchet M (2004) Assessing senescence patterns in populations of large mammals. Anim Biodiv Conserv 27: 47-58.

Garrott RA, Taylor L (1990) Dynamics of a Feral horse population in Montana. J Wildl Manage 54: 603-612.

Ginsberg JR, Rubenstein DI (1990) Sperm competition and variation in Zebra mating-behavior. Behav Ecol Sociobiol 26: 427-434.

Grzimek B (1990) Grzimek’s Encyclopedia of Mammals. New York: McGraw-Hill Publishing Company.

Heise-Pavlov PM, Heise-Pavlov SR, Nelson JE (2009) *Sus scrofa*: Population structure, reproduction and condition in tropical north-eastern Australia. Acta Silv. Lign. Hung 5: 179-188.

Helle E, Kauhala K (1993) Age structure, mortality, and sex ratio of the raccoon dog in Finland. J Mammal 74: 936-942.

Hoogland JL, Foltz DW (1982) Variance in male and female reproductive success in a harem-polygynous mammal, the black-tailed prairie dog (Sciuridae: *Cynomys ludovicianus*). Behav Ecol Sociobiol 11: 155-163.

Hoogland JL (1995) The Black-tailed Prairie Dog: Social Life of a Burrowing Mammal (University of Chicago Press, Chicago).

Houston DB (1982) The northern Yellowstone elk: Ecology and management. New York: Macmillan.

Iossa G, Soulsbury CD, Baker PJ, Harris S (2008) Sperm competition and the evolution of testes size in terrestrial mammalian carnivores. Funct Ecol 22: 655-662.

Jones KE, Bielby J, Cardillo M, Fritz SA, O’Dell J, Orme DL et al. 2009. PanTHERIA: a species-level database of life history, ecology, and geography of extant and recently extinct mammals. Ecology 90: 2648.

Kenagy GJ, Trombulak SC (1986) Size and function of mammalian testes in relation to body size. J Mammal 67: 1-22.

Knight RR, Eberhardt LL (1985) Ecology 66: 323–334.

Koehler GM, Pierce DJ (2005) Survival, cause-specific mortality, sex, and ages of American black bears in Washington state, USA. Ursus 32: 339-347.

Kristiansson H (1990) Population variables and causes of mortality in a hedgehog (*Erinaceous europaeus*) population in Southern Sweden. J Zool 220: 391-404.

Kruuk H, Conroy JWH (1991) Mortality of otters (*Lutra lutra*) in Shetland. J Appl Ecol 28: 83-94.

Hamilton Jr. WJ, Eadie WR (1964) Reproduction in the otter, *Lutra canadensis*. J Mammal 45: 242-252.

Lander RH (1981) A life table and biomass estimate for Alaskan fur seals. Fish Res 1: 55–70.

Leader-Williams N (1988) Reindeer on South Georgia: the ecology of an introduced population. Cambridge: Cambridge University Press.

Loison A, Festa-Bianchet M, Gaillard JM, Jorgenson JT (1999) Age-specific survival in five populations of ungulates: evidence for senescence. Ecology 80: 2539-2554.

Mertens H (1985) Structures de population et tables de survie des buffles, topis et cobs de Buffon au Parc National des Virunga, Zaire. Revue d'Ecologie (Terre et Vie) 40: 33-51.

Moss CJ (2001) The demography of an African elephant (*Loxodonta Africana*) population in Amboseli. J Zool 255: 145-156.

Mueller CC, Sadleir RMFS (1979) Age at first conception in black-tailed deer. Biol Reprod 21: 1099-1104.

Nakagawa N, Ohsawa H, Muroyama Y (2003) Life-history parameters of a wild group of West African patas monkeys (*Erythrocebus patas patas*). Primates 44: 281-290.

Nelson BB, & Chapman JA (1982) Age determination and population characteristics of red foxed from Maryland. Z. Saügetierk 47: 236-311.

Owen-Smith N (1990) Demography of a large herbivore, the greater kudu *Tragelaphus strepsiceros*, in relation to rainfall. J Anim Ecol 59: 893-913.

Packer C, Herbst L, Pusey AE, Bygott JD, Hanby JP, Cairns SJ, Borgerhoff Mulder M (1988) Reproductive success in lions. In Reproductive Success. Edited by Clutton-Brock, T.H. Chicago, University of Chicago Press.

Ramm SA, Parker GA, Stockley P (2005) Sperm competition and the evolution of male reproductive anatomy in rodents. Proc R Soc Lond B 272: 949-955.

Regher EV, Lunn NJ, Amstrup SC, Stirling I (2007) Effects of earlier sea ice breakup on survival and population size of polar bears in Western Hudson Bay. J Wildl Manage 71: 2673-2683.

Reimers E (1983) Mortality in Svalbard reindeer. Holarctic Ecol 6: 141-149.

Sarasa M, Serrano E, Pérez JM, Soriguer RC, Gonzalez G, Joachim J, Fandos P, Granados JE (2010) Effects of season, age and body condition on allocation to testes mass in Iberian ibex. J Zool 281: 125-131.

Sayer JA, Van Lavieren LP (1975) The ecology of the Kafue lechwe population of Zambia before the operation of hydro-electric dams of the Kafue River. Afr J Ecol 13: 9-37.

Setchell JM, Charpentier M, Wickings EJ (2005) Sexual selection and reproductive carreers in mandrills (*Mandrillus sphinx*). Behav Ecol Sociobiol 58: 474-485.

Silva MB, Downing JA (1995) CRC handbook of mammalian body masses. CRC Press.

Schauss ME, Coletto HJ, Kutilek MJ (1990) Population characteristics of wild pigs, *Sus scrofa* in eastern Santa Clara County, California. Calif. Dep. Fish. Game 48: 68-77.

Soulsbury CD, Baker PJ, Iossa G (2008) Fitness costs of dispersal in red foxes (*Vulpes vulpes*). Behav Ecol Sociobiol 62: 1289-1298.

Soulsbury CD (2010) Genetic patterns of paternity and testes size in mammals. PlosOne 5(3): e9581.

Spinage CA (1970) Population dynamics of the Uganda defassa waterbuck (*Kobus defassa* ugandae Neumann) in the Queen Elizabeth Park, Uganda. J Anim Ecol 39: 51-78.

Spinage CA (1972) African ungulates life tables. Ecology 53: 645-652.

Sukumar R, Krishnamurthy V, Wemmer C, Rodden M (1997) Demography of captive Asian elephants (*Elephas maximus*) in Southern india. Zoo Biol 16: 263-272.

Taber RD, Dasmann RF (1957) The dynamics of three natural populations of the deer *Odocoileus hemionus columbianus. Ecology* 38: 233-246.

Toïgo C, Gaillard JM, Festa-Bianchet M, Largo E, Michallet J, Maillard D (2007) Sex- and age-specific survival of the highly dimorphic Alpine ibex: evidence for a conservative life-history tactic. J Anim Ecol 76: 679-686.

Tourmente M, Gomendio M, Roldan ERS (2011) Sperm competition and the evolution of sperm design in mammals. BMC Evol Biol 11: 12.

Waser PM, Elliott LF, Creel NM, Creel SR (1995) Habitat variation and mongoose demography. In Serengeti II: Dynamics, management and conservation of an ecosystem (ed. A. R. E. Sinclair & P. Arcese). Chicago: The University of Chicago Press.

Wigal RA, Chapman JA (1983) Age determination, reproduction, and mortality of the gray fox (*Urocyon cineraoargenteus*) in Maryland, U.S.A*.* Z. Säugetierkunde 48: 226–245.

Wilkinson D, Smith GC, Delahay RJ, Rogers LM, Cheeseman CL, Clifton-Hadley RS (2000) The effects of bovine tuberculosis (*Mycobacterium bovis*) on mortality in a badger. J Zool 250: 389-395.

Wootton, J. T. 1987. The effects of body mass, phylogeny, habitat, and trophic level on mammalian age at first reproduction. Evolution 41: 732-749.
